# Reform in Tuberculosis Work

**Published:** 1917-01-20

**Authors:** 


### REFORM IN TUBERCULOSIS WORK.

### II. The Sanatorium.

By A TUBERCULOSIS WORKER.

To recapitulate: in a previous article there were given detailed instances going to show that the tenure of office of most sanatorium medical residents and superintendents is short and precarious. It now remains to relate the only instance to the contrary within the writer's knowledge. The most prominent characteristic of the gentleman concerned was undoubtedly extreme subserviency. Formerly (although none the worse for that, by any means) he was a pharmacist. If a patient had to be sent away for misconduct, he left the task to the secretary. The well-salaried secretary kept three hunters and was tenant of the institutional grounds, which he farmed for his own profit, the patients' exercise being limited to two narrow paths. The matron, who was head of an enormous number of nurses and maid-servants, filled the notice-boards with injunctions to patients. She had direction of the junior male subordinate staff, the secretary of the senior. The resident medical officer was practically a nonentity. Some considerable part of his daily activity seemed to be swift running to the telephone when the office rang up. Like many other sanatorium residents, he was equal to a good day's work under open-air conditions, but could not have stood the different strain of general practice; and it is by medical men similarly situated that the supply of sanatorium medical workers has largely been kept up, for until the war supervened the salaries given were extremely small. At an epoch when pecuniary circumstances of employment are looked into nearly, one may point to the fact that the financing of the sanatorium movement has been somewhat helped by the cheapness with which the necessary resident medical service has been obtained.

### The System at Fault.

But the main object in citing this success, such as it was, is that in putting it against the failures first given a comparison is obtained which supplies a clue to the main reason why British sanatoria are continually changing their medical residents, and are therefore, by so much, inefficient. For if it be unreasonable to suppose that sanatorium residents are, one and all, shifty good-for-nothings, it is equally unreasonable to set down their fellow-employees as treacherous intriguers and their governing committees as cantankerous tyrants. Where the fault is, is in the system of direction and government of these necessary institutions, necessary for their prophylactic educative effect, and for their adjuvant action on the general health, even were a very potent specific remedy for tuberculosis to be discovered. Here, again, a great deal of well-sounding protestation has been made. The efficiency of a sanatorium, one has often enough heard, depends upon its resident medical man. Sanatorium patients are not as a class easily manageable invalids, susceptible to feminine rule, but mostly such as respond well to a hardy, full-fed, out-of-door life. Moreover, their exercise and rest need to be more rigidly controlled than a schoolboy's. Nevertheless, in almost every large British sanatorium the governing committee has installed the hospital system. Not that one would say anything against that system in its proper place; but only that its proper place is not the sanatorium. The most successful sanatoria—witness, Nordrach, Saranac Lake, Frimley, the Treloar Home—have been those where the real initiative or clinical prestige was supplied by the resident medical head, and where visiting physicans had small or no influence. Nordrach did not possess any regularly employed nurses: a successful British private sanatorium of this type had only one to forty or fifty patients. Where the medical resident is merely a superior sort of house physician, a sanatorium cannot possibly do good work. Nor can the ordinary visiting consultant, who in nine cases out of ten is a general physician, possibly keep abreast of the latest developments in phthisiology, which is fast becoming a specialty.

### The Essential Reform.

The tuberculosis officer may; if he is experienced and studious and above the average in ability. But to give him supervisory powers over the sanatorium superintendent will equally lessen the latter's prestige, with the bad results already mentioned. The present style of matron, who in her hospital days has been accustomed to direct internal affairs altogether, and who has small notion of the frugality, and neglect of mere appearances, sanatorium finances require, unfortunately nearly always gets to loggerheads with the medical resident, who, coming into contact as he does, on ill-defined terms, with all classes connected with the institution—with its patients, its *personnel*, and its patrons—frequently fails to develop the multitude of facets necessary to smooth interaction. Before long he resigns or is asked to resign. But by now most sanatorium committees must have had enough wearisome experience of these veiled mishaps, and be ready to try a different method. Their readiness for change will be increased, too, by the present war-shortage of eligible medical men. The plan here advised is to give those in resident medical charge of the institutions named a position exactly equivalent to that of medical superintendents of asylums for the insane, which by reason of the capacity of their inmates for productive work, and of the necessity for economical management, have much affinity with the large centralised sanatorium of the near future. The sanatorium superintendent should engage (and have the power of dismissal of) every other employee, subject to the approval of a governing committee, on which body he should have a seat and a vote. He should have no medical superior, but be encouraged to invite visits from anyone responsible for a real advance in the knowledge of his subject, especially a foreign worker. Appointments to a sanatorium superintendentship should in future be made as far as possible exclusively from those candidates who have already been assistants at these institutions, just as in the case of asylums. In this way a reasonable average of continuity of tenure, which attends nearly every other medical appointment, would soon also attend sanatorium medical residentship. And the level of clinical attainment at home institutions would rise to that, say, of those of thinly populated Scandinavia: no longer would it be possible for the sobriquet of a British sanatorium situated abroad to be the such-and-such "Sports Club and Home of Rest for Nurses."

It is true that these candid and salutary remarks may offend some vested interests, and antagonise, at first, some readers. They are not—are not meant to be—in the nature of a *placebo*, or filmed over with the soapiness that so commonly obscures comment. They have the rather harsh ring of truth that began to come into use only eighteen months ago. But whether on that account there is less or more profit in them is a question to which we conceive only one answer possible. In the concluding article similar close criticism will be offered of the work of the tuberculosis officer.

